# Development of Control Algorithms for an Adaptive Running Platform for a Musculoskeletal Rehabilitation System

**DOI:** 10.3390/s25216667

**Published:** 2025-11-01

**Authors:** Artem Obukhov, Andrey Volkov

**Affiliations:** Laboratory of VR Simulators, Tambov State Technical University, Tambov 392000, Russia; didim@eclabs.ru

**Keywords:** adaptive running platforms, musculoskeletal rehabilitation, control functions, human tracking systems

## Abstract

An essential component of modern musculoskeletal rehabilitation systems is treadmills of various sizes, the control of which may rely either on manual adjustment of treadmill speed, fixed for the entire training session, or on automatic regulation based on analysis of the user’s movements and velocity. The aim of this study was to experimentally compare the control functions of an adaptive treadmill designed for musculoskeletal rehabilitation and to assess the influence of the hardware configuration and tracking systems on user stability and the smoothness of transient processes. Two running platforms (of different lengths, one equipped with handrails and one without), two tracking systems (virtual reality trackers and a computer vision system using the MediaPipe Pose model), and three control functions—linear, nonlinear, and proportional-integral-derivative (PID)—were investigated. A set of metrics with both metrical and physiological interpretability was proposed (including positional stability, duration and amplitude of transient processes in position and velocity, subjective assessment, and others), all integrated into a single quality control criterion. This study presents extensive experimental research comparing various designs of adaptive running platforms and tracking systems, exploring the relationships between the available working area length and user comfort, and determining the optimal parameters for the selected control functions. The optimal control function was identified as the linear law for the tracking system based on virtual reality trackers and the PID function for the computer-vision-based tracking system. The conducted experiments made it possible to formulate recommendations regarding the minimum permissible working area length of treadmill platforms and the selection of tracking systems and control functions for musculoskeletal rehabilitation systems. The obtained results are of practical relevance for developing adaptive rehabilitation simulators and creating control algorithms that ensure smooth and stable treadmill motion, thereby enhancing user comfort, efficiency, and safety during musculoskeletal rehabilitation exercises.

## 1. Introduction

Statistics show that up to 80% of the population has various musculoskeletal problems, such as lower back pain, neck pain, and postural disorders [1,2]. Therefore, organising rehabilitation and restoring human motor functions is a pressing and important task for society. Musculoskeletal rehabilitation comprises several key areas [3].

The first class of musculoskeletal rehabilitation systems can be described as platforms or complexes with end-effector devices or robotic systems, meaning that they fix the user at a single endpoint (typically at the ankle) [4]. This simplifies the adaptation of the system to the individual characteristics of the user, but it creates difficulties in manual correction of body parts during rehabilitation due to insufficient fixation. Representatives of this class include products such as the Gait Trainer GTI, Haptic Walker, Rutgers Ankle, Ankle Rehabilitation roBOT (ARBOT) and others [5].

The second class involves the use of various exoskeletons. In such systems, the patient’s legs or body are fully actuated through fixation at multiple points. Examples of such systems include Lokomat, Lokohelp, LOwer-extremity Powered ExoSkeleton (LOPES), Active Leg EXoskeleton (ALEX), Active Ankle-Foot Orthosis (AAFO), Berkeley Lower Extremity Exoskeleton (BLEEX), and others [6]. The principle of user movement can be organised either with treadmills (in this case, the lower part of the exoskeleton at the foot is absent) or without them (the foot then also moves with the help of the exoskeleton). This class of systems requires adaptation to the body or lower limb area, i.e., adjustment or regulation of segment lengths using movable parts or other approaches due to significant differences in leg and limb lengths among patients. The disadvantages of such systems also include the frequent need for several operators to perform rehabilitation, high cost, complexity, and restriction of the patient’s freedom of movement.

An important component of second-class systems is a controlled treadmill used both at the initial stage of rehabilitation, in combination with robotic manipulators to support and move their lower limbs, and at the final stage, when the patient moves independently on the treadmill with or without support [7]. This aspect highlights the high relevance of implementing adaptive running platforms that adjust to the speed and specifics of human movement to ensure maximum comfort and safety.

Previous studies have demonstrated the high effectiveness of adaptive running platforms controlled by intelligent algorithms based on data from sensors or cameras in the field of professional training [8]. Subsequent research aimed to study the applicability of these results in musculoskeletal rehabilitation with the aim of improving the quality of human interaction with adaptive running platforms. The following research questions were addressed in this study to deal with the issue:

- Analysis and comparison of various hardware implementations of adaptive running platforms used for musculoskeletal rehabilitation;
- Study of various methods of tracking and positioning a person on a running platform, as well as methods for controlling the platform, taking into account the specifics of tracking systems;
- Conducting extensive experimental studies comparing different designs of adaptive running platforms, as well as studying the correlation between the available length of the platform’s working area and user comfort;
- Developing recommendations for the minimum acceptable dimensions of the running platform’s working area, tracking systems, and control functions for musculoskeletal rehabilitation systems.

Solving these tasks will improve the quality of musculoskeletal rehabilitation systems by improving adaptive running platforms, their control algorithms, and approaches to tracking user movements. The novelty of the research lies in developing and comparing new algorithms for controlling adaptive running platforms based on machine learning and computer vision technologies, and studying patterns of human interaction with platforms with different hardware parameters.

In contrast to previous studies on adaptive treadmills, which focused either on individual control algorithms (including linear, nonlinear, neural network, or zonal approaches) without considering different hardware implementations, or on various hardware configurations without an in-depth analysis of the algorithms, the present study integrates both aspects. The novelty of this work lies in the comprehensive experimental evaluation of three types of commonly used control algorithms (linear, nonlinear, and PID) on two treadmill configurations featuring different tracking systems. Furthermore, an integral control quality criterion is introduced, simultaneously accounting for positional stability, smooth acceleration and deceleration, adaptability, and user comfort. Thus, the present study proposes a new methodology for the combined assessment and adaptation of treadmill control functions, including recommendations for selecting optimal settings and the minimally required system parameters (such as the critically important aspect of the minimum sufficient platform length).

This paper is structured as follows: The first section outlines the relevance and purpose of this study. The second section analyses existing research into adaptive running platforms and their control systems. The third section examines the architecture of the control system for the developed running platform, the proposed control functions and the procedure for conducting experimental studies to compare them and determine the optimal parameters. The final section presents a comparison of the various control functions and the selection of the optimal parameters, as well as an analysis of user movement on the platform. The article concludes with a discussion of the results and final remarks.

## 2. Related Work

### 2.1. Analysis of Research in the Field of Adaptive Running Platforms

As is well known, various types of running platforms can be used for movement within virtual reality, including sliding, push-type, spherical, and active platforms [8]. In the field of musculoskeletal rehabilitation, it is necessary to take into account the specifics of this process: the platform user must perform movements that are as close as possible to natural walking, experience varying degrees of load from their body weight, and the surface on which the user moves must be flat and not create additional obstacles. Thus, the use of the following types of platforms is not possible in this subject area:

- Sliding platforms based on a special concave hemispherical coating that allows a person to move along them using safety equipment [9]: unnatural movement on a slippery surface, inability to organise correct and natural walking mechanics.
- Pressure platforms based on displacement in the required direction by tilting the platform after pressure from the user [10]: no movement mechanisms other than rolling, restriction of the user’s movement area, applicability only for testing reaction mechanisms and balance control.
- Suspended platforms, similar in design to sliding platforms, using a suspension mechanism to hold the user instead of a safety mechanism: inability of the user to feel the physical load from the movement process, freely rotate the body, or move quickly across the platform [11].
- Spherical platforms in which a person moves inside a large-diameter rotating sphere, constantly remaining at the lowest point of the sphere: not applicable in musculoskeletal rehabilitation, negatively affect the vestibular apparatus [12].

The use of all types of passive platforms in musculoskeletal rehabilitation is unjustified due to technical limitations and the physical condition of the user. On the other hand, active platforms capable of adjusting the speed of movement are successfully used in many musculoskeletal rehabilitation systems. Adaptability, i.e., the ability to adjust to the user’s movement patterns, is an important feature of modern systems. Not all active platform designs are equally applicable to musculoskeletal rehabilitation. The analysis showed that the following types of active platforms have critical disadvantages when used in this area:

- Platforms based on a ball grid (lattice), where each element of the grid moves in the opposite direction to the user’s movement (e.g., Cybercarpet [13]): high inertia, lack of physical loads typical for natural movement.
- Multifaceted or multi-section platforms, in which each section moves independently of each other with a fixed centre [14]: discomfort when moving at the junction of two sections, unrealistic when moving in a straight line due to the transition from moving to stationary sections of the platform.

Therefore, active controlled single, dual or omnidirectional platforms based on the principle of treadmills are the most applicable. Given the need to connect controlled manipulators to move the user’s legs, there is no need for omnidirectional platforms, as this would only complicate the design and increase the system’s cost. The ability to move in both directions (forward and backward) depends on the rehabilitation method, but in most musculoskeletal rehabilitation systems, the treadmill belt only moves in one direction.

### 2.2. Analysis of Approaches to Tracking Individuals on Platforms

As previous studies have shown, tracking a person on the platform is one of the most important tasks. This is due to the possibility of sudden, unplanned movements by the user, as well as the possibility of a fall, which would require the platform to stop immediately.

Experience gained in tracking human movements [15] has shown that ultrasonic and optical sensors, as well as feedback systems, are not very suitable for this task. When using ultrasonic sensors, it is possible to frequently receive erroneous data due to human displacement and low sensor accuracy. Optical sensor systems require the use of a larger number of receivers and transmitters, between which a person must pass in order to fix their position. In addition, they are quite sensitive to vibrations (from the active platform). Finally, systems based on feedback analysis may have a large margin of error due to the conversion of analogue signals to digital. Thus, the two most suitable tracking systems are wireless sensors (trackers) designed for virtual reality kits and computer vision systems.

Virtual reality trackers allow for high-precision tracking (with an error margin of less than 0.01 m) within the visibility range of base stations (up to 9 × 9 m). However, when leaving the visibility range of base stations or when they are blocked, signal loss from sensors is possible, leading to incorrect data. In addition, the size of the trackers limits the areas where they can be mounted and their total number. In the field of musculoskeletal rehabilitation, given the specific nature of users’ conditions and the exercises they perform, a number of tracking systems are inapplicable or have certain drawbacks [16]: the use of trackers is justified when the musculoskeletal rehabilitation system includes a virtual reality helmet. The computer vision system is sensitive to the appearance of strangers in the field of view, as well as poor lighting conditions. Nevertheless, these systems have significant advantages that allow us to focus on their comparison when organising an adaptive running platform control system: high accuracy of virtual reality trackers [17] and the ability to track more than 30 points (depending on the neural network model used for body recognition) without additional mounted devices [18].

Existing experience in the field of human tracking shows that computer vision technologies are actively used to analyse and classify human posture and gait [19], which is highly significant for identifying emergency situations occurring on the platform, as well as assessing the user’s status during rehabilitation.

Computer vision systems are actively used to analyse running technique in research and clinical conditions [20], as they provide easy-to-configure, affordable and reasonably accurate results. In this study, at a sufficiently high speed (up to 3.33 m/s), the error in determining the angle of flexion of the lower limbs did not exceed 11 degrees on the model trained by the authors and 7.5 degrees on the OpenPose model. The study [21] compared the kinematics of the lower limbs and the spatio-temporal parameters of gait obtained using a single-camera markerless method with parameters obtained using a marker-based motion tracking system. A high correlation was found between the two approaches, and the average difference relative to the marker method was: the values for stride and step length were 5.08 cm (4.42%) and 3.96 cm (6.72%), respectively; the value for walking speed was 0.10 m/s (10.87%). Although the obtained results are promising, the authors emphasise that the accuracy may be insufficient for clinical applications.

The choice of the MediaPipe Pose model (based on the BlazePose architecture) for the computer vision system is driven by a compromise between accuracy and performance. Alternative models are currently being actively developed, including the YOLO family, OpenPose, and various proprietary solutions [22,23,24]. OpenPose has demonstrated high accuracy in the aforementioned studies; however, its real-time application is limited due to substantial computational requirements. In contrast, MediaPipe Pose is optimised for real-time operation on standard computers and mobile devices, achieving approximately 30 frame per second (FPS) without the need for a powerful GPU [25]. This factor was decisive in selecting the tracking system. It is worth noting that the MediaPipe model used covers 33 key points (including hands and feet) and is specifically designed for augmented reality and motion analysis applications where performance is critical.

Despite having fewer tracking points than computer vision technologies, trackers remain a promising tool for monitoring body movements due to their accuracy and absolute positioning. In [26], three trackers attached to the thigh, knee, and ankle were used to measure the trajectory of linear knee movement and circular ankle movement. The measurement accuracy for various sports exercises was high enough to enable visualisation of a limb or even the entire body using inverse kinematics. Thus, the use of six trackers allows for the monitoring of the lower limbs, while additional trackers on the torso (for example, one or two on the back and abdomen) enable the assessment of the person’s position and movement speed on the treadmill. Two additional trackers are justified to improve the reliability of user position tracking (through position averaging and drift control). However, in the simplest case, a single tracker is sufficient to obtain information about the user’s displacement along the treadmill. A similar positive result is observed in paper [27], where Vive Trackers were used to collect complete kinematic data of the lower limb joints. The authors obtained continuous tracker trajectories for all segments of the lower limb. Analysis of the repeatability of the results showed similar repeatability with advanced motion tracking systems.

The effectiveness of tracker-based tracking systems has also been confirmed in monitoring other parts of the body. For example, in [28], trackers were used to analyse 12 different upper limb movements using neural networks to reconstruct complete kinematics. Considering the accuracy of the initial data from virtual reality sensors, such reconstructions are certainly possible and allow the complete body model to be reconstructed with low error based on a limited dataset (5–7 points) [15,29].

Alongside markerless approaches, the “gold standard” for human motion tracking remain marker-based optoelectronic systems (such as the Vicon), which provide high spatial–temporal accuracy for multiple body segments. According to the validation studies [30], the Vicon demonstrates millimetre-level positioning errors in static conditions and low dynamic errors when correctly calibrated and configured; typical sampling rates of 100 Hz or higher allow detailed 3D kinematic reconstruction and computation of joint angles, moments, and spatio-temporal gait parameters. These findings are confirmed both by targeted performance tests and by numerous studies where the Vicon serves as the reference system for dataset generation and clinical gait analysis automation.

Comparative analyses between marker-based and markerless systems in systematic reviews and applied studies have shown that, when properly configured, markerless approaches achieve acceptable accuracy in evaluating certain spatio-temporal indicators and sagittal-plane kinematics, although they still lag behind the Vicon in absolute angular/positional precision and robustness to occlusions and lighting conditions [31]. Therefore, the Vicon and similar systems are justified where clinical precision and full 3D kinematics are essential, whereas markerless approaches are a sufficient alternative for engineering applications where accessibility, simplicity, and scalability are prioritised.

In the context of the present study, namely, the task of treadmill belt speed regulation based on user position, the use of systems such as the Vicon is impractical. Achieving the objective does not require highly precise tracking of all body segments or high-accuracy 3D reconstruction. It is sufficient to reliably estimate the longitudinal coordinate with an error of a few centimetres at a frequency of ≥30 Hz and a latency of about 100 ms. These requirements are met by VR trackers and the cost-effective markerless system based on MediaPipe. The use of open and accessible technologies instead of proprietary high-cost equipment significantly reduces implementation barriers without compromising the system’s primary goal—stable and safe treadmill control [30].

The analysis of tracking systems confirms the applicability of computer vision systems and virtual reality trackers in the implementation of treadmill control systems. Unlike previous studies [8], when using computer vision, it is planned not to be limited by a small number of points (previously, only the position of the feet was analysed), but to use of the entire human body as source data.

### 2.3. Analysis of Existing Research in the Field of Platform Control Systems

Evaluating existing control functions and research conducted in this area, it is necessary to take into account the following features of their implementation:

- Linear algorithms remain the simplest, most reliable and transparent in their operation, without affecting the performance of the control system, and are successfully used for unidirectional active platforms with speeds of up to 3 m/s [32]; since the algorithm adjusts the belt speed proportionally to the displacement that has already occurred, without analysing the displacement trend or its rate of change, it cannot predict and inevitably introduces control latency. This is a common drawback of proportional control approaches [33].
- Nonlinear algorithms can be more flexible in a number of situations, for example, during sudden or slow changes in speed [34]. In addition, nonlinearity can arise due to the specifics of the tracking method (for example, camera distortion introduces nonlinearity even for the linear control function);
- Proportional-derivative (PD) or proportional-integral-derivative (PID) control laws [35,36], widely used in various control systems, including the field of musculoskeletal rehabilitation;
- Neural network algorithms have been actively developed in recent years, and trained models are becoming increasingly flexible in their operation, allowing for the assessment of the user’s gait and condition to identify abnormal situations [37] and the speed of human movement [38].

Previous studies have shown that researchers often adhere to a specific list of known functions: linear, nonlinear, and those based on proportional–differential and proportional-integral-differential [8]. A comparative analysis of these functions in two tracking systems (virtual reality trackers and computer vision systems) showed the following results: Six functions were compared: linear, nonlinear, zonal, detailed zonal, proportional–differential, and neural network, as well as two fundamentally different positioning methods based on trackers and machine vision. The experiments revealed the ambiguity of the function selection: in the tracker-based positioning method, the best results were shown by the nonlinear and detailed zonal functions developed by the authors; in the machine vision-based positioning method, the best results were shown by the linear, nonlinear, and proportional–differential functions. The application of the neural network control function showed unstable results, although it has certain prospects.

## 3. Materials and Methods

### 3.1. Architecture of the Adaptive Running Platform Control System

**Figure 1:**
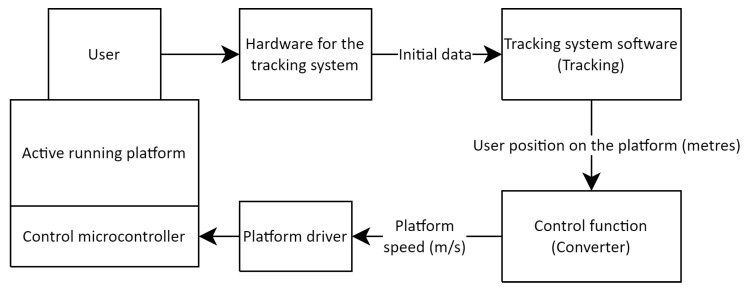
The general architecture of the adaptive running platform control system.

In the first stage of this study, it is necessary to examine the general architecture of the adaptive running platform control system, as well as the hardware used for the platforms in this work (Figure 1).

The system includes: the user on the treadmill; a position tracking system (in two configurations—with VR trackers and base stations or based on a computer vision system); a tracking data processing module (Tracking) that computes positional metrics; a converter module (Converter) implementing the control algorithm by calculating the required belt speed based on the user’s position; and the treadmill driver, which receives the target speed and adjusts the engine speed, thereby changing the belt movement speed. The entire chain forms a closed loop: the user’s position is continuously tracked and used to correct the treadmill speed in real time, ensuring adaptation to the walking pace.

**Figure 2:**
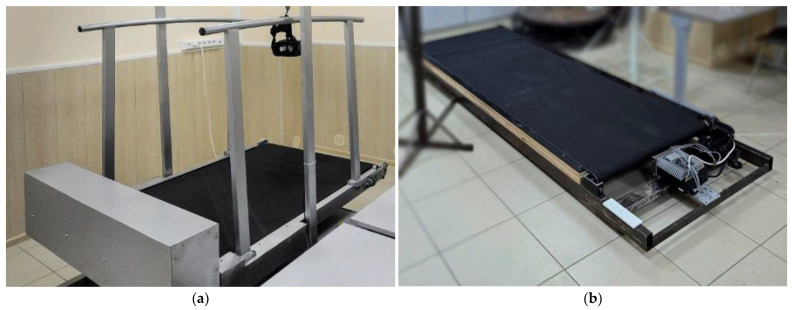
Appearance of treadmill platforms: (**a**) standard length with adjustable angle; (**b**) extended length without angle adjustment.

The architecture under consideration is based on an active running platform, which can have different functionalities and implementation specifics. This study considers two types of platforms: a standard length platform with adjustable angle (No. 1) and an extended platform without angle adjustment (No. 2), which are presented in Figure 2. An analysis of the platforms’ characteristics is presented in Table 1.

The main distinguishing features between the platforms are their dimensions (the length of the track, which directly affects the working area) and maximum speed, which can provide a radically different user experience. The presence of handrails on platform No. 1 also makes a significant difference to the user experience. Platform No. 1 was previously used to compare different tracking systems and control functions, after which it was found that its length might be insufficient, leading to the creation of platform No. 2, which has different characteristics. The greater length of the platform allows the impact of this parameter on user comfort to be assessed, while the absence of handrails and angle adjustment is due to the orientation of platform No. 2 for musculoskeletal rehabilitation.

**Figure 3:**
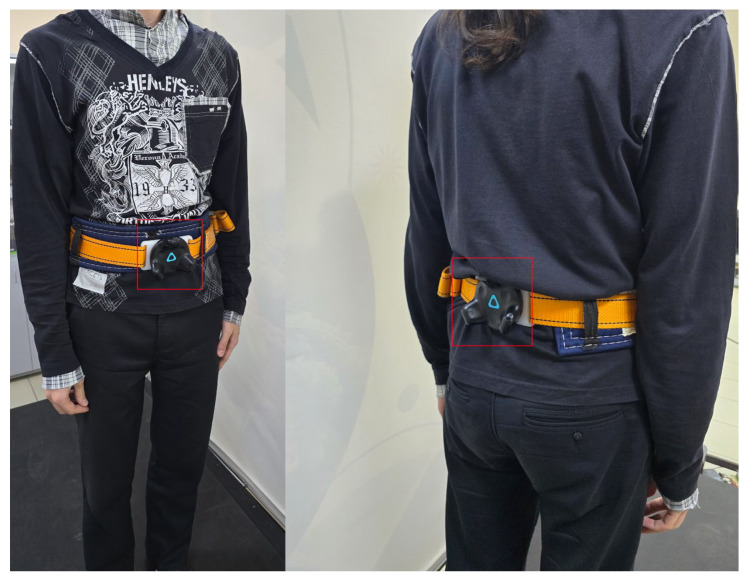
Tracker mounting scheme.

The choice of tracking system, which includes both hardware selection and software application and refinement, is of great importance in solving the task of adaptive platform management. It is essential to focus on the hardware aspects. In accordance with the above analysis, two tracking systems will be used in the study:

S1: a set of virtual reality sensors HTC Vive Trackers, a recording frequency of 60 Hz. The tracker mounting scheme is shown in Figure 3. Two trackers of the system are attached to the person’s waist: one in front (on the belt around the abdomen) and the other symmetrically at the back (on the lower back). Such placement ensures stable position tracking: even if one tracker temporarily loses connection or becomes occluded, the other continues transmitting coordinates.

S2: a computer vision system based on a camera with a resolution of 1080 p and a recording frequency of 30 Hz. The camera is installed at a distance of 1.5 m from the edge of the treadmill.

When selecting tracking systems, the following acceptability criteria were applied for the control task: longitudinal positioning error up to 5 cm (2–3% of the total treadmill length); update frequency of 30 Hz; and total latency of 100 ms. Configuration S1 (VR) confidently meets these conditions; S2 (MediaPipe) complies with them under proper camera setup and controlled recording conditions (lighting, absence of occlusions), as demonstrated by previous and external studies [21,39].

The details of the implementation of the tracking system software are presented in Section 3.2. To summarise the work of this module, it should be noted that the tracking system software receives and processes the source information (from trackers or cameras), after which it generates the position of the person on the platform in the metric system (values from 0 to 2.5 m relative to the start of the track).

The obtained position is then processed by the control function in the Converter module. This module implements a number of different control functions, which are described in Section 3.3 and Section 3.4. The functions are united by the uniformity of the returned speed value, expressed in metres per second. This value is transmitted to the adaptive running platform driver in accordance with the previously developed operating algorithm [40]. The speed is then sent to the platform’s control microcontroller, which directly sets the desired speed. The described cycle of data collection, transmission and processing is repeated multiple times every second, ensuring continuous regulation of the platform’s speed.

**Figure 4:**
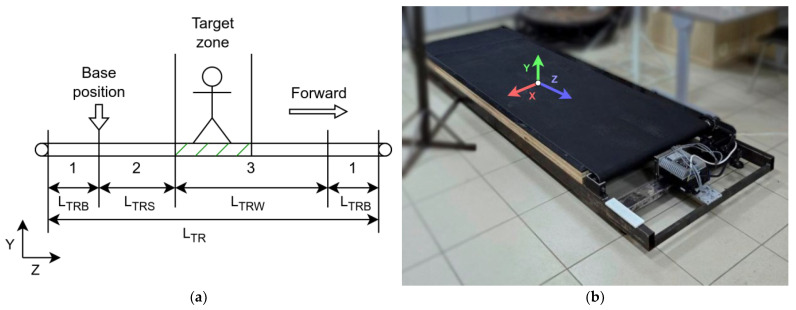
Positioning on the running platform: (**a**) division of the running platform into zones; (**b**) location of the coordinate system.

The running platform belt with the total length $L_{TR}$ can be conditionally divided into several zones (Figure 4a): the boundary zone (1) with the length $L_{TRB}$, necessary for compliance with safety rules, this zone provides a margin from the edges of the belt to the boundaries of the zone in which the user must move. The safe zone (2) with the length $L_{TRS}$; the running platform stops when the user is in this zone. The working zone (3) with the length $L_{TRW}$; when the user remains in this zone, the running platform moves according to the selected control function. Figure 4b shows the direction of the coordinate axes on the actual platform (platform No. 2).

The size of zone (1) is 0.3 m, and zone (2) is determined experimentally and is equal to 0.1 m. Zone (1) is sufficiently large to compensate for the inactive edge of the treadmill and to allow the user to stand comfortably near the boundary. Zone (2) represents a short section of the belt that triggers the platform to stop when entered. Various values were tested during the system setup phase to determine this length. The chosen size (0.1 m) ensures a rapid treadmill stop as soon as the user steps beyond the main working area. Considering Zone (1) of 0.3 m, this provides a total of 0.4 m, which is sufficient for the idle and stop zones, even taking into account the inertia of the treadmill belt.

### 3.2. Tracking System Software Implementation

To implement the tracking module for a person on a treadmill platform, the previously developed software was refined and upgraded [8]. The implementation for each type of tracking system is discussed below.

The S1 system software (based on trackers) is based on the use of the OpenVR library. Virtual reality trackers within the visibility range of base stations transmit information about the tracker’s position (absolute coordinates along the X, Y, Z axes in metres) and its rotation angles (yaw, pitch, roll) in a coordinate system tied to the location of the base stations in the room (Figure 4b). At the start of the control system, calibration is performed, i.e., the initial position of the trackers is fixed as the starting point relative to which the position is subsequently calculated (Figure 4a). This allows the trackers to be strictly positioned in the working area of the track relative to the initial safe position. This position can be either a certain starting point of the platform or its centre in the case of a sufficiently large length (in this case, zones 2 and 3 in Figure 4a are mirrored relative to the base point, which allows the running platform to be used for movement in two directions if necessary).

The software ensures reliable operation by using positioning via two trackers attached to the user’s back and stomach. There are three possible situations:

- Both trackers are tracked normally: the positions of the two trackers are averaged to obtain the average body position;
- One tracker is detected: only its position is taken, adjusted for the user’s body width, which eliminates sudden jumps in position;
- No trackers are found: an error is returned.

In addition, the software includes the function of monitoring the status of trackers: if the distance between two trackers exceeds the initial distance by L% (for example, 50%), then the so-called tracker drift is recorded. Drift is understood to be a smooth and continuous distortion of the tracker’s position caused by interference from base stations or partial obstruction (by clothing, a hand), which is difficult to detect by software if there is only one tracker.

Since trackers determine their position in the metric coordinate system, the position obtained after the above checks is forwarded to the Converter module.

Further, the functioning of the computer vision-based tracking system software will be examined. Unlike the previous study [8], the MediaPipe Pose model is used as the neural network for human body recognition, allowing not only tracking of the user’s legs, but also the entire body model. This allows additional protection modules to be implemented based on this information, for example, to recognise the moment of a fall for emergency shutdown of the platform [41]. In the future, having a full-body model will make it possible not only to detect a fall but also to recognise signs of loss of balance (for example, abnormal forward or backward leaning of the torso), incorrect movement (sideways walking), or characteristic changes in gait associated with fatigue (shortened step length, foot dragging). Upon detecting such patterns, the system could suggest ending the session for the patient’s safety. It should be noted that some of these safety functions can also be implemented using trackers by analysing their position and tilt angles in three-dimensional space.

During software development, it was necessary to take into account the specifics of the MediaPipe Pose neural network, which returns the coordinates of 33 body points in relative values from 0 to 1 within a captured frame from the camera. This led to the need to create a separate module for converting relative coordinates into metric coordinates with a number of additional procedures.

**Figure 5:**
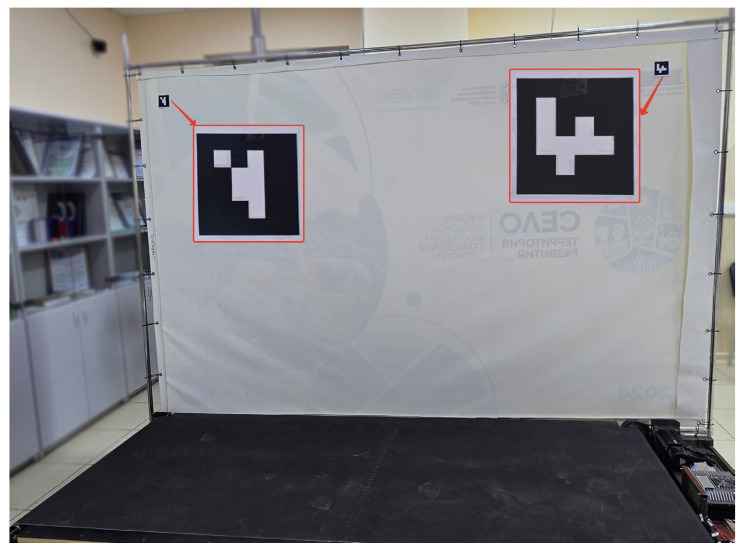
The marker placement scheme for the computer vision system.

Firstly, camera distortion is corrected using a standard procedure to obtain a calibration matrix [42]. Secondly, the background image contains two markers whose positions are recorded and stored in the configuration file. An example of the markers is shown in Figure 5; the markers are attached to the upper corners of the background canvas.

Each time the system is started, these markers are recognised again, and if their position deviates significantly from the specified position, it can be concluded that the camera has shifted from its original position, which can lead to unstable and incorrect operation of the entire control system. Finally, the module for converting MediaPipe Pose normalised coordinates to the metric system, based on a pre-calibration mechanism, has been implemented:

1. The camera is placed perpendicular to the running platform so that its working area fits entirely within the width of the frame, and the person fits within the height of the frame. The position is fixed using the markers mentioned earlier.
2. The user stands in the far left position of the platform in the centre, and the points of their waist are fixed as the starting position.
3. The user stands at the far right of the platform in the centre, and the points on their waist are fixed as the final position.
4. During the recognition of the human body, its current position is converted using the following formula:

(1) $$U^{0}=L_{TR}\cdot \frac{\left(u_{x}-s_{x}\right)}{e_{x}-s_{x}},$$

where $L_{TR}$ is the length of the platform in metres;

$u_{x}$ is the user’s current position in the frame along the X-axis, obtained from MediaPipe Pose based on two points on the waist;

$s_{x}$, $e_{x}$ are the saved initial and final positions;

5. In the case of a sufficiently long running platform, it is possible to modify the conversion formula to obtain the metric position of a person relative to the centre of the platform rather than its initial position, as presented in Formula (1) [43]:

(2) $$U_{c}=L_{TR}\cdot \frac{\left(u_{x}-s_{x}\right)}{e_{x}-s_{x}}-\frac{L_{TR}}{2}.$$

Within the framework of this study, for the computer vision system, it is planned to use the latter option presented in Formula (2). Since virtual reality trackers can also be calibrated at the centre of the treadmill, the metric position from both systems, which will hereafter be denoted as U, will be determined within a unified system of relative coordinates.

Thus, both tracking systems return the position of the person on the platform, expressed in metres.

### 3.3. Implementation of Adaptive Running Platform Control Functions

Based on the results obtained earlier [8] comparing various control functions, the following functions implemented in the Converter module will be used in this study. Each function takes the user’s position U (in metres) relative to the initial calibration point as input.

The $F_{L}$ linear function determines the speed of the platform based on the position of the person U relative to the boundary of the safe zone:

(3) $$F_{L}(U)=\frac{\left(U-L_{TRS}\right)V_{P}}{k_{l}-L_{TRS}},$$

where $L_{TRS}$ is the size of the safe zone in metres;

$k_{l}$ is the set size of the working zone, in metres. Its length is equal to the sum of the lengths of zones 2 and 3 of the platform (Figure 4a): $k_{l}=L_{TRS}+L_{TRW}$. The selection of the working zone size is described in Section 4.2;

$V_{P}$ is the maximum speed of the platform, m/s.

The $F_{N}$ nonlinear function is based on a smoother change in speed and may vary depending on the value of the $k_{\alpha }$ coefficient:

(4) $$F_{N}(U)={\left(\frac{U-L_{TRS}}{k_{l}-L_{TRS}}\right)}^{k_{\alpha }}V_{P},$$

It has been previously established that acceptable values for the $k_{\alpha }$ parameter are in the range $0.3\leq k_{\alpha }\leq 0.7$. The selection of optimal parameter values is described in Section 4.2.

The proportional-integral-derivative (PID) function $F_{PID}$ is based on the PID control law and uses information about the user’s displacement relative to the previous measurement, as well as their speed (derived from this displacement):

(5) $$F_{PID}(U_{t+\Delta t},t+\Delta t)=k_{p}(U_{t+\Delta t}-L_{TRS})+k_{i}\int_{0}^{t+\Delta t}\left(U_{t+\Delta t}-U_{t}\right)+k_{d}\left(\frac{U_{t+\Delta t}-U_{t}}{\Delta t}\right),$$

where $U_{t+\Delta t}$, $U_{t}$—are the current and previous positions of the user at times t+Δt and t, respectively;

$F_{PID}(U_{t+\Delta t},t+\Delta t)$ is the new velocity value for time (t+Δt) at position $U_{t+\Delta t}$ of the person; 

$k_{p},k_{i},k_{d}$ are correction coefficients that determine the influence of the proportional, integral, and derivative components of the $F_{PID}$ function. The selection of coefficient values is described in Section 4.2.

The presented functions performed successfully during testing of platform No. 1 using various tracking methods, which makes it relevant to verify their effectiveness on the new platform No. 2. The control formulas used correspond to established practice [33,34,35].

### 3.4. Choosing Metrics for Assessing Management Quality

The following designations will be introduced:

$z_{abs}=Z_{abs}(t)$ is the instantaneous absolute position of the user on the platform (in metres) at any given moment in time t.

$z_{0}=Z_{abs}(0)$ is the absolute position of the user at the start of the experiment (at time t=0).

$z=Z(t)=Z_{abs}(t)-z_{0}$ is the instantaneous displacement of the user on the platform at any given moment in time relative to the starting position $z_{0}$.

$z_{mean}=median(z)$ is the median value of the user’s displacement for the entire duration of the experiment.

To evaluate the quality of the control functions, a set of metrics was developed to assess various aspects of the control functions. The set includes the following metrics:

$R_{ZM}$ is the median value of the user’s displacement during the experiment (metres, lower is better), $R_{ZM}=z_{mean}$.

$R_{ZD}$ is the range of change in the user’s position during the experiment (metres, lower is better). It is calculated as the difference between the maximum and minimum positions of the user during the entire experiment: $R_{ZD}=\max(z_{abs})-\min(z_{abs})$.

$R_{ZMD}$ is the value of the stability interval for the user’s position (metres, lower is better). This metric measures the range of fluctuations in the user’s position in steady state; a lower value indicates a more stable position. The steady state interval is determined as follows:

1. The user’s position at the initial moment in time $z_{0}$ is taken as the initial point;
2. The DZ(t) characteristic is calculated as the modulus of the difference between the user’s current position and the initial position: $DZ(t)=\left|Z_{abs}(t)-z_{0}\right|$;
3. The value of the 90th percentile of DZ(t) is taken as the stability interval: $R_{ZMD}=P_{90}(DZ(t))$.

$R_{ZTO}$ is the time it takes to reach the operating mode based on the user’s position (seconds, lower is better). This metric characterises the duration of the transition process between the idle state and the steady state of the control system. In a simplified form, this metric is defined as the time from the start of the treadmill speed adjustment to the moment when the patient first becomes almost motionless relative to the treadmill for at least two seconds.

$R_{ZAO}$ is the maximum deviation of the user’s position along the Z-axis (along the treadmill) relative to the established mean position in the steady-state mode during the transition period is measured in metres (lower is better). This metric characterises the amplitude of the transition process.

The $R_{ZTO}$ and $R_{ZAO}$ metrics are calculated as follows. For the user position function Z(t), time intervals are determined in which the user’s position can be considered stationary according to the following algorithm:

1. The time interval Δt = 0.5 sec is considered, and the user’s position is considered stationary in the middle of this interval if the restriction $z_{mean}-R_{ZMD}\leq z\leq z_{mean}+R_{ZMD}$ is satisfied for more than 80% of the time in this interval. The threshold value of 80% was chosen as a compromise based on a preliminary data analysis. At higher thresholds (for example, 90–100%), the criterion becomes too strict and may ignore the actual stabilisation due to isolated noise peaks; at significantly lower thresholds (50–60%), on the contrary, there is a risk of falsely considering an unstable section as steady. The processing of our experimental signals has shown that the 80% value effectively filters out noise, allowing the reliable identification of the moment when a stationary state is reached.
2. The interval under consideration is shifted by 0.02 s at each iteration. The first interval with a stationary position whose duration exceeds 2 s is selected.
3. The value of the $R_{ZTO}$ metric is calculated as the difference between the start time of the experiment and the start time of the interval found in step b (i.e., the time of reaching the operating mode).
4. The value of the $R_{ZAO}$ metric is calculated as the difference between the maximum value of the user’s displacement during the time it takes to reach operating mode and the median position of the user. If the maximum position value is less than $z_{mean}$, the value 0 is used as $R_{ZAO}$, i.e., negative values of this metric are discarded: $R_{\mathrm{ZAO}}=\max(0,{max}_{t\in [0,R_{ZTO}]}Z(t)-z_{mean})$.

$R_{STO}$ is the time required to reach operating mode based on the speed of the belt (seconds, lower is better);

$R_{SAO}$ is the maximum value of the surge in the speed of the belt relative to the established average speed in operating mode during the time required to reach operating mode (m/s, lower is better);

The $R_{STO}$ and $R_{SAO}$ metrics are calculated in a similar way, but instead of user displacement, the speed of the belt is considered.

The values of the metrics $R_{ZTO}$, $R_{ZAO}$, $R_{STO}$, and $R_{SAO}$ determine the magnitude and duration of the transition process that occurs at the start of movement. The faster the platform reaches a steady state in terms of speed and position, the shorter the delay in adapting to the external environment (user actions). Large speed surges and significant fluctuations in the user’s position on the platform are undesirable, as they can cause discomfort or loss of balance for the user.

$R_{ZFMEAN}$ is the mathematical expectation of the amplitude of «fast» oscillations of the user’s position (metres, lower is better). In simplified terms, the metric can be understood as the mean absolute position error along the Z-axis in the steady-state mode (after the system has reached its established operating regime). Such oscillations may be associated with noise generated by the position tracking system or the platform control system. The amplitude of «fast» oscillations is calculated as the modulus of the difference between the upper and lower envelopes of the Z(t) function. It is calculated as follows:

1. For the Z(t) function, the upper (based on local maxima) and lower (based on local minima) envelopes $Z_{H}(t)$ and $Z_{L}(t)$ are constructed;
2. The value of the oscillation amplitude function is calculated: $Z_{A}(t)=\left|Z_{H}(t)-Z_{L}(t)\right|$;
3. The value of the mathematical expectation of the $Z_{A}(t)$ function is taken as $R_{ZFMEAN}$.

$R_{ZFSMAE}$ is the average absolute error of the $Z_{F}(t)$ function relative to $Z_{S}(t)$, (metres, lower is better). The value of $Z_{F}(t)$ is calculated by applying the 5th-order Butterworth low-pass filter with a cutoff frequency of 0.5 Hz to the Z(t) function. The value of $Z_{S}(t)$ is calculated using a similar filter with a cutoff frequency of 0.1 Hz. This indicator reflects the degree of discrepancy between the user’s instantaneous position and the average trajectory of their movement. It allows for an indirect assessment of the comfort level of the control system; the lower the MAE, the more predictable the user’s movement relative to the average pace. It is calculated as follows:

1. The $Z_{F}(t)$ function is calculated by filtering the original Z(t) function with the 5th order Butterworth low-pass filter with a cutoff frequency of 0.5 Hz (which eliminates noise generated by the tracking system);
2. The $Z_{S}(t)$ function is calculated by filtering the original Z(t) function with the 5th order Butterworth low-pass filter with a cutoff frequency of 0.1 Hz (which smooths out small movements of the user while walking);
3. The $Z_{FSA}(t)$ function is calculated as the modulus of the difference between $Z_{F}(t)$ and $Z_{S}(t)$: $Z_{FSA}(t)=\left|Z_{F}(t)-Z_{S}(t)\right|$;
4. The value of $R_{ZFSMAE}$ is taken as the mathematical expectation of the $Z_{FSA}(t)$ function.

$R_{G}$ is the average subjective assessment of the quality of the control function provided by the participants in the experiment (a dimensionless value, higher is better). It is measured on a scale from 1 (very bad) to 10 (very good).

The metrics used to assess quality have different dimensions and different directions for improving the indicator. To obtain a general control error criterion, it is necessary to bring them to a single dimension. For this purpose, the standard normalisation procedure will be used:

(6) $$R_{n}=\frac{R-R_{base}}{R_{max}-R_{base}},$$

where the following abbreviations are used:

$R_{n}$ is the normalised value of the metric;

R is the initial value of the metric;

$R_{max}$ is the maximum value of this metric in all experiments;

$R_{base}$ is the base value of the metric. In this case, 0 is used as the base value for normalisation, rather than the minimum value of the metric, since the values of all metrics in the integral criterion should tend towards zero.

When calculating the integral control error criterion $R_{Q}$, it is necessary to invert the direction of measurement of all metrics that need to be maximised. For example, the $R_{G}$ metric: $R_{G}^{\prime }=1-R_{G}$. Thus, all metrics used can be taken into account in the weighted sum $R_{Q}$ according to the formula:

(7) $$R_{Q}=\sum_{i=1}^{N}w_{i}R_{i},$$

where $w_{i}$ is the weight coefficient of the i-th metric, N is the total number of metrics.

The weighting coefficients used are not equal, since an expert-based ranking of metrics according to their priorities can be performed (Table 2): safety, followed by stability, and finally comfort. Thus, the metrics directly related to the safety and stability of the control system (amplitudes and durations of position/velocity oscillations, deviation indicators in the steady-state mode at the 90th percentile level) are assigned higher weights; the subjective user assessment is included with a moderate weight, allowing comfort to be taken into account without reducing the priority of safety. During the experimental studies (Section 4.5), sensitivity will be tested (the influence of weight variations on the final ranking of control functions).

Combining metrics into a single integral criterion allows several aspects of control efficiency to be taken into account at once: speed, stability, smoothness, adaptability, and comfort.

### 3.5. Procedure for Conducting Experimental Research

The experimental research will consist of two stages. The preliminary stage will determine the optimal parameters for each of the selected control functions. The main stage will compare various control functions and tracking systems, as well as test a number of hypotheses concerning the influence of various features of the running platform on the nature of the user’s movements.

It should be noted that in this study, the maximum speed of both platforms was limited to $V_{P}=2$ m/s for the safety of the test subjects. This limitation was due to the fact that the study involved a large number of test subjects who had not previously participated in experiments on these treadmills.

It is worth mentioning that during the experiments particular attention was paid to the safety of the participants. In addition to speed limitations on each platform, a number of additional safety measures were implemented. During the trials, an operator was constantly present nearby, ready to immediately press the emergency stop button if a participant experienced balance issues or any other problems. The control algorithm also included an automatic protection mechanism: when the user moved into the extreme areas of the treadmill (beyond the working zone), the system stopped automatically, preventing a fall. Platform No. 1 was equipped with handrails and a safety harness that participants could use to maintain balance. All participants also underwent thorough instruction to understand the operating principles of the treadmill. Thanks to these measures, all measurements were carried out without any incidents, confirming the sufficient safety of the selected experimental protocol.

The preliminary stage is conducted on platform No. 2 with a small group of test subjects (5 people) using a tracking system based on virtual reality trackers (which shows maximum accuracy [16]). For each of the selected control functions, a range of acceptable parameter values was determined, from which the optimal ones must be selected:

- For the linear function $F_{L}$, the parameter $k_{l}$ (working section length) is varied, the value is selected from the following set: 0.5, 0.75, 1, 1.25, and 1.5 m;
- For the nonlinear function $F_{N}$, the parameters $k_{l}$ (working section length) and $k_{\alpha }$ (nonlinearity coefficient) are varied. $k_{l}$ values are selected in the same way as for the linear function, except for the value 1.25 m (to reduce the number of tests), $k_{\alpha }$ values are selected from the set: 0.3, 0.4, 0.6; the values of $k_{\alpha }$ were chosen based on the analysis of the results of the previous study [8]: values that are too small (less than 0.3) make the function almost indistinguishable from the linear one, whereas excessively large values (close to 1) make the platform’s response overly smooth and sluggish. The range of 0.3–0.6 covers the characteristic range of nonlinearity, from pronounced (α = 0.3) to moderate (α = 0.6).
- For the PID control function, the parameters $k_{i}$ (integral coefficient) and $k_{d}$ (derivative coefficient) are varied. Each of the parameters is selected from the set: 0.1, 0.5, 1, 2. The value of the $k_{p}$ parameter (proportional coefficient) is set equal to 2, which corresponds to the linear control function with $k_{l}=1.1$ when the maximum platform speed is limited to $V_{P}=2$ m/s. This limitation was introduced for safety reasons, as the speed of 2 m/s approximately corresponds to a very fast walk or light jog and is sufficient to simulate active gait. Higher speeds (up to 5 m/s and above), although technically feasible, are typical for running and sprinting and therefore are not the target of musculoskeletal rehabilitation; moreover, they pose an increased risk of falling, especially for untrained users.

For each set of parameters, the corresponding experiment configuration is entered: 5 options for linear, 12 options for nonlinear, and 16 options for PID function. Each configuration includes three trials lasting 60 s. The interval of 60 s was chosen to allow the participant to reach a steady walking mode and remain in it long enough for data collection. Observations have shown that the stabilisation of speed and position is achieved within the first 5–15 s after the start of movement, while the remaining ~45–55 s provide a representative sample of data on steady motion. Shorter trials (for example, 20–30 s) would yield insufficient data for reliable conclusions, whereas significantly longer intervals (several minutes) could lead to participant fatigue and a change in gait pattern. The triple repetition of each test condition was also determined as a compromise: three repetitions make it possible to average the results and reduce the effect of random deviations (for example, those caused by brief distraction), without excessively tiring the participant. The test subject must smoothly reach a comfortable speed and maintain this speed throughout the experiment. After the defined time has elapsed, the treadmill automatically stops and data recording stops. After completing three trials, the user gives an overall subjective assessment of this configuration based on their comfort level while walking. A total of 165 experiments were conducted in the preliminary stage, and 495 records were obtained.

During the main stage of the experiment, the following issues are considered:

- Comparison of the performance of selected control functions at the best parameters for each function, selected at the preliminary stage (conducted on platform No. 2).
- Comparison of the platform control system using tracking methods S1 and S2 under identical conditions (conducted on platform No. 2).
- Assessment of the user’s ability to independently maintain a constant position on the running platform while the platform is moving at a given constant speed under various conditions:
  ○ The user’s ability to move in the centre of the platform without using aids or landmarks (conducted on platform No. 2);
  ○ the user’s ability to move within a specified area measuring 50 × 50 cm in the centre of the platform, marked on the treadmill surface using a projector (conducted on platform No. 2);
  ○ the user’s ability to move in the centre of the platform while holding onto the handrails (performed on platform No. 1);
- Comparison of positional accuracy and other metrics characterising gait uniformity when the automatic control system is operating and when the user is moving at a constant speed.
- Assessment of the impact of the presence of handrails on the operation of the automatic platform control system (performed on platforms No. 1 and No. 2 using the S1 tracking system and the $F_{L}$ control function).

The structure of the main stage is similar to that of the preliminary stage of the study. Three trials, each lasting 60 s, are also conducted for each experiment configuration. The following experiment configurations are presented in the main stage:

- A total of 7 configurations using automatic speed control: 6 on platform No. 2, 2 for each of the control functions described above: using the S1 and S2 tracking systems, and one configuration on platform No. 1 using the S1 system and the $F_{L}$ control function. In this series of experiments, the user’s sequence of actions completely repeats the preliminary stage.
- A total of 12 configurations in which the running platform moves at a fixed speed: without markings (platform No. 2), with markings (platform No. 2), with handrails (platform No. 1) at speeds of 0.5 m/s, 1 m/s, 1.5 m/s, as well as at a speed chosen by the test subject as the most comfortable. In this series of experiments, the user must try to maintain their initial position on the platform. The user is informed in advance of the start and end of the experiment by a three-second countdown, and the platform accelerates to the set speed and stops smoothly with a constant acceleration of 1 m/s^2^.

**Figure 6:**
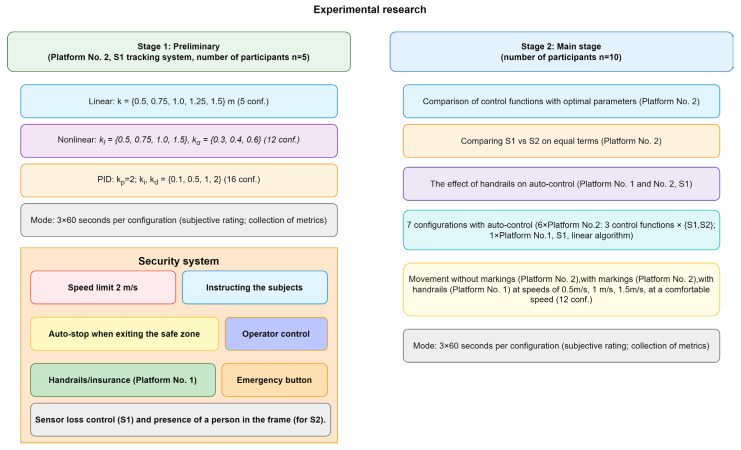
The framework of the research.

To better illustrate the proposed approach, a diagram of the research methodology was developed (Figure 6). It shows the two stages of the experiments: the preliminary stage (selection of the optimal parameters of the control functions) and the main stage (comparison of various system configurations), as well as the implemented safety measures.

A larger group of test subjects, totalling 10 people, was involved in the main stage of the study. A total of 190 experiments were conducted during the main stage, and 570 records were obtained. The group consisted of male participants aged 22.5 ± 3.2 years, with a mean height of 179 ± 5.23 cm.

Thus, 355 experiments were conducted, using sequential numbering from 1 to 355.

## 4. Results

### 4.1. Analysis of the User’s Movement on the Treadmill

The process of user movement on a treadmill in various operating modes is examined. This section illustrates the analysis of the collected data, including the process of calculating the metrics presented above.

**Figure 7:**
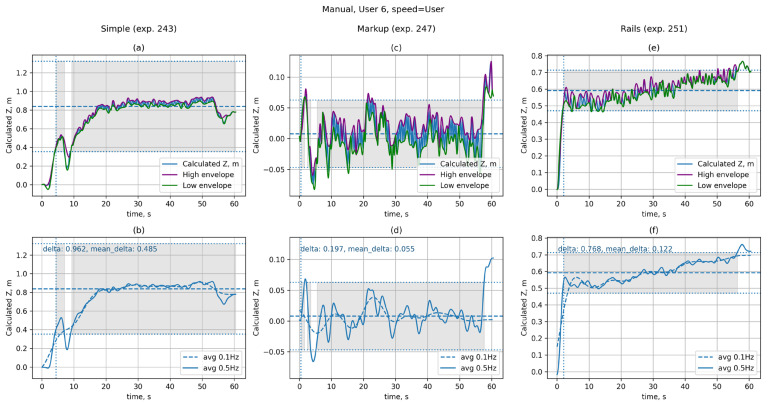
User displacement from the initial position during walking at constant speed: (**a**) without auxiliary means, raw data; (**b**) without auxiliary means, averaged data; (**c**) with treadmill surface marking, raw data; (**d**) with treadmill surface marking, averaged data; (**e**) with handrail support, raw data; (**f**) with handrail support, averaged data.

Figure 7 shows graphs of user deviation from the starting position during platform movement at a specified speed under various conditions: without the use of assistive devices (Simple, Figure 7a,b), with the use of markings (Markup, Figure 7c,d), and with the use of handrails (Rails, Figure 7e,f). These experiments were selected for illustration, as their data are representative of each of the cases under consideration. The numbers 243, 247, and 251 are provided to specify the entries in the database and to enable subsequent identification of the measurement.

The graphs show data obtained from experiments 243, 247, and 251. The top row (Figure 7a,c,e) displays raw data (Z(t) function) for the first trial, while the bottom row (Figure 7b,d,f) shows the data after processing with the 5th order Butterworth low-pass filter with a cutoff frequency of 0.5 Hz (solid line) and 0.1 Hz (dashed line) that is the $Z_{F}(t)$ and $Z_{S}(t)$ functions, respectively. The horizontal dashed line is drawn at the median position value ($z_{mean}$). The horizontal dotted lines mark the boundaries of the stationarity interval in terms of position $\left[z_{mean}-R_{ZMD},z_{mean}+R_{ZMD}\right]$, and the grey fill indicates the areas in which the position value lies within the stationarity interval. The vertical dotted line indicates the end time of the transition process ($R_{ZTO}$).

As can be seen from the graphs for experiment 243 (Figure 7a,b), without additional reference points, the user shifts significantly from the starting position ($R_{ZD}=0.962$ m), despite the desire to maintain the initial position on the platform. The relatively high value of $R_{ZMD}=0.485$ m also indicates low stability of the user’s position during the experiment. The use of handrails in experiment 251 (Figure 7e,f) increases the overall stability of the position ($R_{ZMD}=0.122$ m), although at the beginning of the movement the user also shifted significantly from the starting position ($R_{ZD}=0.768$ m). The presence of markings on the belt in experiment 247 (Figure 7c,d) allows the user to maintain the specified position ($R_{ZD}=0.197$ m) with good accuracy and also significantly increases the overall stability of the position ($R_{ZMD}=0.055$ m).

**Figure 8:**
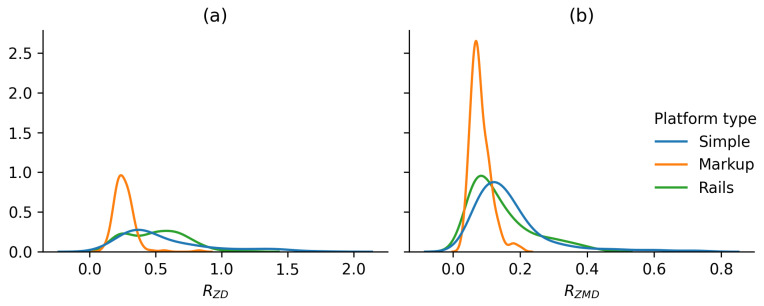
Distribution of metric values at constant platform speed: (**a**) $R_{ZD}$; (**b**) $R_{ZMD}$.

As can be seen from the distribution of the corresponding metrics shown in Figure 8, this behaviour is typical for most users.

**Figure 9:**
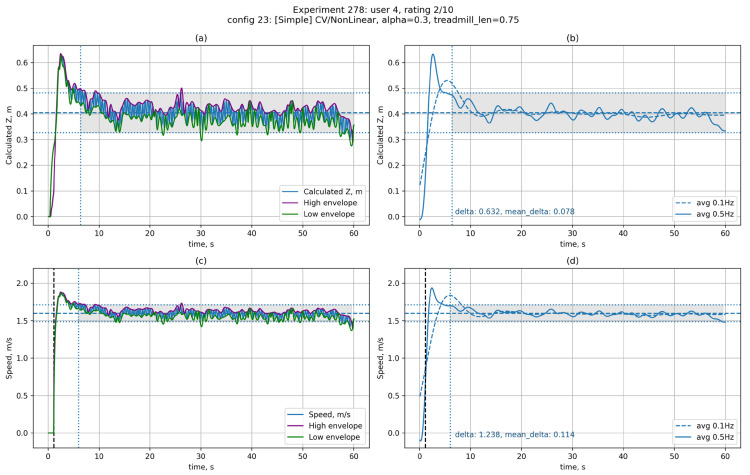
User movement in automatic treadmill control mode: (**a**) displacement from the initial position, raw data; (**b**) displacement from the initial position, averaged data; (**c**) treadmill belt speed, raw data; (**d**) treadmill belt speed, averaged data.

Figure 9 shows graphs of the user’s deviation from the starting position (Figure 9a,b) and the platform’s speed (Figure 9c,d) when the user is moving in automatic mode (experiment 278: tracking system S2, $F_{N}$ control function).

The left side (Figure 9a,c) shows raw data, while the right side (Figure 9b,d) shows filtered data. The auxiliary lines are labelled similarly to Figure 7, with an additional vertical dotted line indicating the start of movement (only on the velocity graph 9c,d). The graphs clearly show a spike at the start of movement, which is characteristic of a nonlinear control function. It is also possible to note the fairly high stability of the user’s position ($R_{ZMD}=0.078$ m), comparable to the use of markings on the belt.

### 4.2. Selecting Optimal Parameters for Control Functions

**Figure 10:**
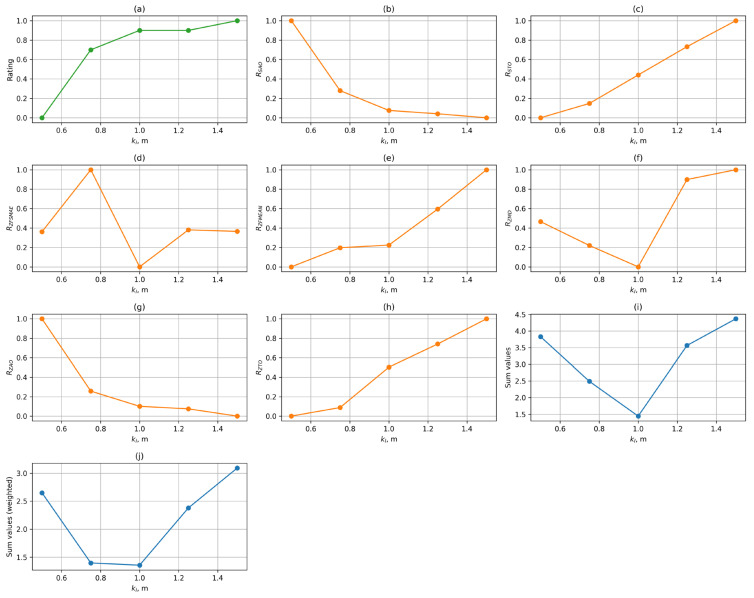
The metric values for the linear control function: (**a**) $R_{G}$; (**b**) $R_{SAO}$; (**c**) $R_{STO}$; (**d**) $R_{ZFSMAE}$; (**e**) $R_{ZFMEAN}$; (**f**) $R_{ZMD}$; (**g**) $R_{ZAO}$; (**h**) $R_{ZTO}$; (**i**) sum of metrics without weighting coefficients; (**j**) weighted sum of metrics.

**Figure 11:**
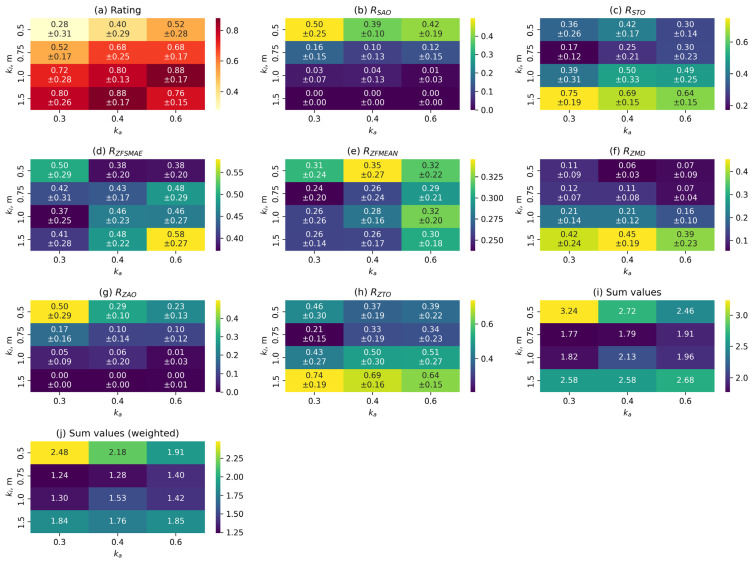
The metric values for the nonlinear control function: (**a**) $R_{G}$; (**b**) $R_{SAO}$; (**c**) $R_{STO}$; (**d**) $R_{ZFSMAE}$; (**e**) $R_{ZFMEAN}$; (**f**) $R_{ZMD}$; (**g**) $R_{ZAO}$; (**h**) $R_{ZTO}$; (**i**) sum of metrics without weighting coefficients; (**j**) weighted sum of metrics.

**Figure 12:**
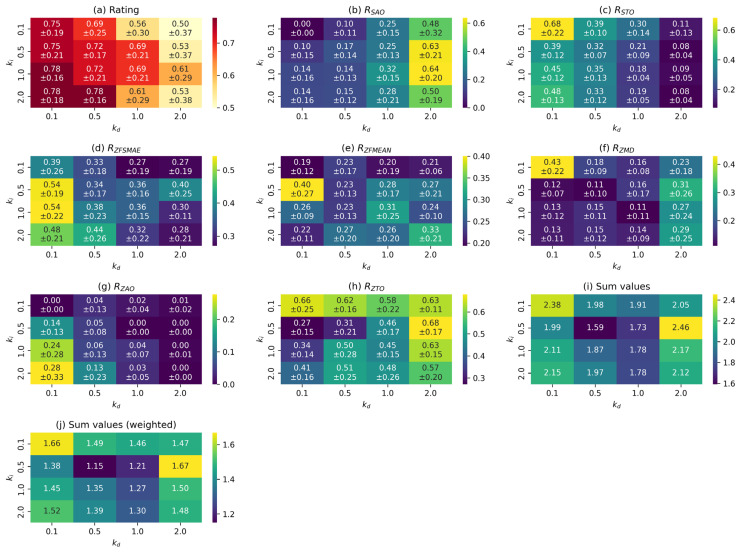
The metric values for the PID control function: (**a**) $R_{G}$; (**b**) $R_{SAO}$; (**c**) $R_{STO}$; (**d**) $R_{ZFSMAE}$; (**e**) $R_{ZFMEAN}$; (**f**) $R_{ZMD}$; (**g**) $R_{ZAO}$; (**h**) $R_{ZTO}$; (**i**) sum of metrics without weighting coefficients; (**j**) weighted sum of metrics.

At the preliminary stage of the study, an optimal set of parameters was determined for each control function. For this purpose, the values of the metrics described in Section 3.4 were calculated based on the collected data, and a summary table of the results was compiled. The results of processing this data are presented in diagrams in Figure 10, Figure 11 and Figure 12.

Figure 10 shows graphs of the dependence of the median normalised values of the metrics described above on the value of the $k_{l}$ parameter for the $F_{L}$ control function. The last two graphs (Figure 10i,j) show the values of the simple sum and the weighted sum of the metrics, taking into account the weighting coefficients. Metrics that follow the «more is better» rule are marked in green, while those that follow the «less is better» rule are marked in orange and blue. The final integral criterion must also be minimised.

As can be seen from the graphs, increasing the length of the working section has a positive effect on users’ subjective assessment. Increasing $k_{l}$ also reduces the amplitude of the transition process, with speed surges virtually absent from a length of 1 m onwards, but at the same time, the duration of the acceleration transition process increases. The indicators characterising gait stability and positional accuracy have complex nonlinear dependencies. The best value for the linear function is $k_{l}=1$ m. This is justified by the fact that in Figure 10j, the working area of 1 m yields the minimum value of the weighted sum of indicators for the linear control function, which demonstrates the optimality of the selected parameter.

Figure 11 shows heat maps of the dependence between the normalised values of the metrics described above and the values of the $k_{l}$ and $k_{\alpha }$ parameters for the $F_{N}$ control function. The red colour palette indicates metrics with values that need to be maximised, while the yellow-green palette indicates metrics with values that need to be minimised. For the metrics where this calculation is applicable, the mean values and their standard deviations are presented.

As can be seen from the graphs, increasing the length of the working segment has a similar effect on the characteristics of the transition process as when using the linear control function. Increasing the $k_{\alpha }$ coefficient also leads to a decrease in its amplitude and an increase in duration, but has a significantly smaller effect than $k_{l}$. Position holding accuracy decreases with an increase in $k_{l}$ and increases slightly with an increase in $k_{\alpha }$. The indicators characterising gait stability have complex nonlinear dependencies. The best set of values for the nonlinear function is $k_{l}=0.75$ m and $k_{\alpha }=0.3$.

Figure 12 shows similar heat maps for the $F_{PID}$ control function. As can be seen from the graphs, most metrics have complex nonlinear dependencies on parameter values. The best set of values is $k_{i}=0.5$ and $k_{d}=0.5$. As mentioned earlier, the value of the $k_{p}$ parameter (proportional coefficient) is set to 2. The optimality of the selected values is due to the fact that this combination corresponds to the lowest weighted sum of criteria (Figure 12j), i.e., it minimises the integral efficiency criterion.

**Figure 13:**
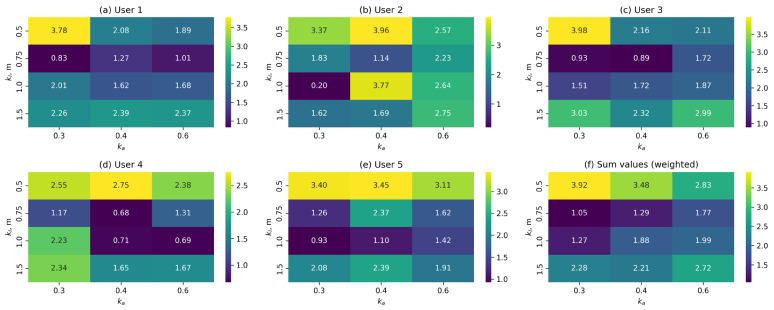
The sum of the metric values for the nonlinear control function for each user: (**a**–**e**) for users 1–5; (**f**) average values across all users.

It should be noted that the sets of parameters obtained for each control function are the best when considering the average results for the entire group of test subjects. However, for each individual user, the best set of parameters will not necessarily coincide with the average results for the entire selection. Figure 13 shows heat maps of the weighted sum of metrics for the $F_{N}$ control function, calculated separately for each user (Figure 13a–e), as well as the average result for comparison (Figure 13f).

As can be seen from the graphs, the best values only coincide with the average result for two out of five users (users 1 and 3). For users 2 and 5, the best result is close to the average. For user 4, however, the overall distribution of ratings and the range of best results differ significantly from those of the other users.

**Figure 14:**
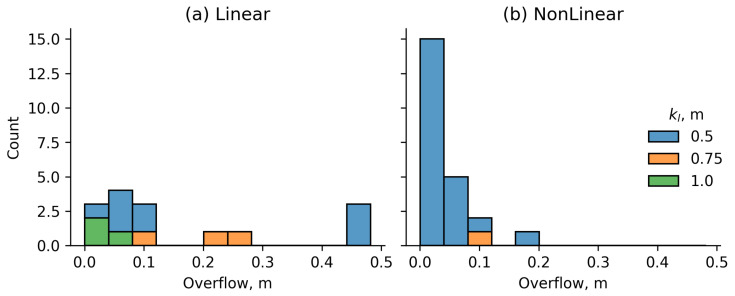
The distribution of the number of user exits beyond the working area: (**a**) for the linear control function; (**b**) for the nonlinear control function.

Additionally, cases were considered in which the user moved beyond the boundaries of the control workspace during movement. When the user leaves the workspace, it means that the control function has failed to perform its task. During the preliminary stage of the experiment, a total of 38 cases (7.7%) of users leaving the working area were recorded, 31 (6.2%) of which occurred when the working area was 0.5 m. Figure 14 shows the distribution of cases depending on the length of the workspace and the control function.

Thus, it can be concluded that the working area of 0.5 m is insufficient for the normal functioning of the automatic control system. For the $F_{N}$ control function, the minimum permissible value of $k_{l}$ is 0.75 m, and for the $F_{L}$ control function, it is recommended to use a working area of at least 1 m. Taking into account the previously defined sizes of the boundary zone, 0.3 m long (used on both sides of the working area), and the safe zone, 0.1 m in length, the minimum recommended treadmill length is 1.7 m. When using a nonlinear control function, the treadmill length can be reduced to 1.45 m, while for PID control a length of 1.8 m is recommended.

### 4.3. Comparison of Control Functions and Tracking Systems

In the main stage of the experiment, a comparison of various control functions was performed using the best parameters selected for each function in the preliminary stage. A comparison of tracking systems S1 and S2 was also performed at this stage.

**Figure 15:**
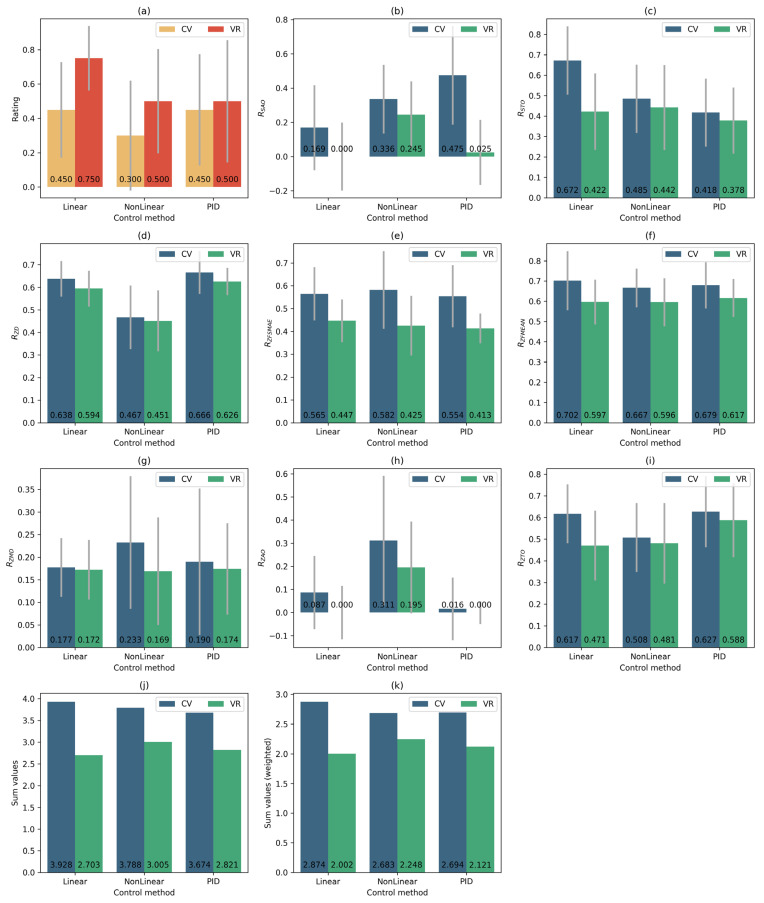
Comparison of different control functions: (**a**) $R_{G}$; (**b**) $R_{SAO}$; (**c**) $R_{STO}$; (**d**) $R_{ZD}$; (**e**) $R_{ZFSMAE}$;(**f**) $R_{ZFMEAN}$; (**g**) $R_{ZMD}$; (**h**) $R_{ZAO}$; (**i**) $R_{ZTO}$; (**j**) sum of metrics without weighting coefficients; (**k**) weighted sum of metrics.

Figure 15 shows comparative diagrams for the median normalised values of metrics when using different tracking systems and control functions. The red colour scale is used for metrics that need to be maximised. The green-blue colour scale is used for metrics that need to be minimised. In Figure 15, the metrics for which higher values indicate better performance (and thus should be maximised) are shown in a red colour scale, whereas the metrics for which lower values indicate better performance (to be minimised) are depicted in a green–blue colour scale. This distinction highlights which criteria benefit from larger values and which from smaller ones. As can be seen from the composite plot, some indicators represent errors and should be as small as possible (smaller values indicate better accuracy), whereas others reflect efficiency, which, on the contrary, should be increased (larger values correspond to better performance).

To verify the statistical significance of differences between the control functions and tracking systems, a non-parametric analysis based on the Mann–Whitney U test was conducted. This approach allows for the correct evaluation of distribution differences even with a small sample size and without the assumption of normality. Pairwise comparisons were performed for all combinations of control functions—Linear, NonLinear, and PID—separately for the VR tracker system (S1) and the MediaPipe system (S2). The analysis was carried out for all main metrics.

The Mann–Whitney test results showed the following (Table 3). For the VR system (S1), statistically significant differences were observed between the linear and nonlinear functions (*p* = 0.0132) and between the linear and PID functions (*p* = 0.0108), whereas the difference between the nonlinear and PID functions was not statistically significant. For the CV system (S2), the differences were less pronounced: the *p*-values of 0.1595 for Linear/NonLinear and 0.7427 for Linear/PID did not reach the significance threshold (*p* < 0.05). However, for the NonLinear/PID pair (*p* = 0.0351), a moderately significant difference was observed.

According to the integral rating $R_{Q}$, the differences between controllers in the VR system reached a high level of statistical significance (*p* < 0.001), confirming that the structure of the control function has a reliable impact on regulation quality. For the CV system, the effect was weaker (*p* ≈ 0.1–0.2), which corresponds to the overall increase in data variance when using this type of tracking.

The temporal metrics demonstrated a statistically significant advantage of the linear controller in the VR system compared with the nonlinear and PID controllers (*p* < 0.05), whereas the spatial metrics proved to be less sensitive to the type of control function.

The following conclusions can be drawn from the obtained results:

- The S1 tracking system shows higher results than the S2 system when using the same set of parameters for each control function. Moreover, the advantage is observed not only in the values of the integral criterion, but also in the values of each individual metric.
- Within a single tracking system, all used control functions show approximately equal results. The best result for the S1 tracking system, with a difference of 5.9% relative to the second result, is shown by the $F_{L}$ linear control function. For the tracking system S2, the best result is shown by the nonlinear control function $F_{N}$. The remaining functions demonstrate slightly lower performance (less than 1% difference from the second-best result).

**Figure 16:**
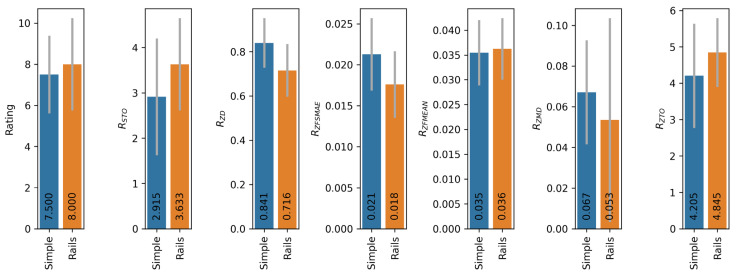
Median values of metrics for platforms No. 1 and No. 2.

Additionally, the impact of handrails on the operation of the automatic platform control system was assessed. For this purpose, identical series of tests were conducted on platforms No. 1 (Rails) and No. 2 (Simple) using the S1 tracking system and the $F_{L}$ control function. During the tests on platform No. 1, users held onto the handrails throughout the experiment. Figure 16 shows comparative diagrams of median metric values without normalisation. The $R_{ZAO}$ and $R_{SAO}$ metrics are excluded from consideration because their values are zero in both cases (no significant spikes are observed at the beginning of the movement).

The obtained data show that the presence of handrails on the running platform slightly increases the stability of the user’s position and also slightly increases subjective comfort (higher rating). At the same time, the duration of the transition process at the beginning of the movement increases. In general, the presence of handrails has a slight positive effect on the operation of the automatic control system.

### 4.4. Analysis of User Movement When the Platform Moves at a Constant Speed

**Figure 17:**
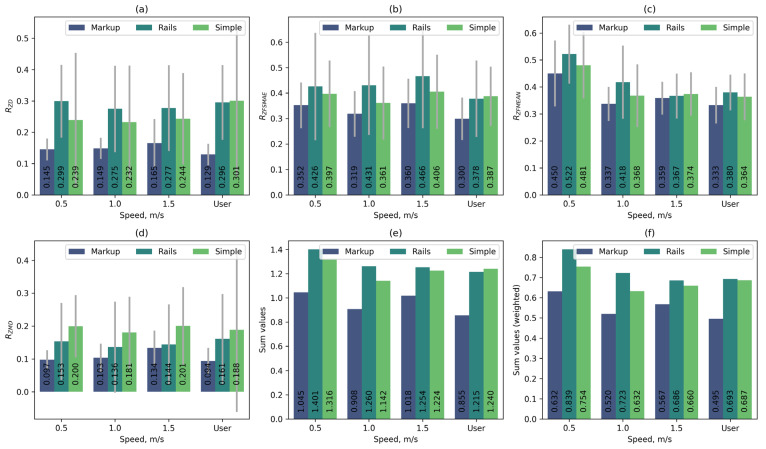
Metrics for user movement at constant platform speed: (**a**) $R_{ZD}$; (**b**) $R_{ZFSMAE}$; (**c**) $R_{ZFMEAN}$; (**d**) $R_{ZMD}$; (**e**) sum of metrics without weighting coefficients; (**f**) weighted sum of metrics.

In addition to comparing automatic control functions, a series of measurements were taken during the main stage of the experiment at constant platform speeds: 0.5 m/s, 1 m/s, 1.5 m/s, and at the speed selected by the user. The tests were conducted on three different platform variants: without the use of auxiliary landmarks on platform No. 2 (Simple), with the target zone projected onto the belt on platform No. 2 (Markup), and with the use of handrails on platform No. 1 (Rails). During movement, users tried to maintain a constant position on the platform. Based on the results of these tests, the metrics $R_{ZD}$, $R_{ZMD}$, $R_{ZFMEAN}$, and $R_{ZFSMAE}$ were calculated. Figure 17 shows the normalised median values of the calculated metrics for all test variants.

**Figure 18:**
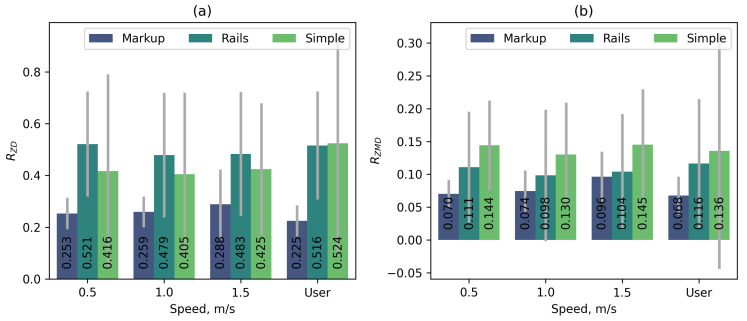
Metrics for manual mode without normalisation: (**a**) $R_{ZD}$; (**b**) $R_{ZMD}$.

Figure 18 additionally shows the median values of the $R_{ZD}$ and $R_{ZMD}$ metrics without normalisation for all variants of constant platform speed.

As can be seen from the graphs, the use of handrails has a minor effect on user movement. Despite the fact that handrails increase stability (lower $R_{ZMD}$ metric value), based on the combination of metrics, the use of handrails does not provide a significant improvement but shows worse results than movement without handrails on platform No. 2. However, projecting the target area onto the treadmill belt significantly improves the user’s stability (on average, 1.75 times according to the $R_{ZD}$ metric and 1.8 times according to the $R_{ZMD}$ metric).

**Figure 19:**
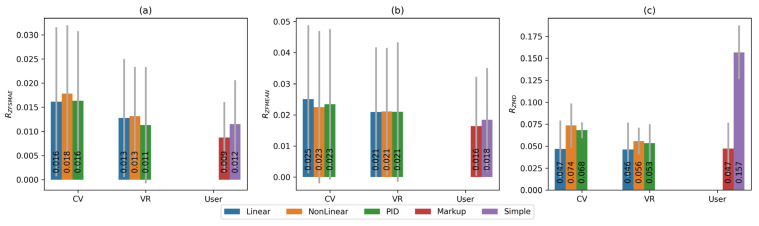
Comparison of metrics in manual and automatic modes: (**a**) $R_{ZFSMAE}$, (**b**) $R_{ZFMEAN}$, (**c**) $R_{ZMD}$.

The comparison of position maintenance accuracy and gait uniformity was also carried out while the automatic control system was operating and the user was walking at a constant speed. Figure 19 shows comparative diagrams of the average values of the $R_{ZFMEAN}$, $R_{ZFSMAE}$, and $R_{ZMD}$ metrics without normalisation.

The graphs show that the user’s gait characteristics in manual and automatic modes are comparable in terms of the $R_{ZFMEAN}$ and $R_{ZFSMAE}$ metrics, and in terms of position stability ($R_{ZMD}$), the selected automatic control functions are comparable to the use of markings on the belt and significantly exceed the user’s ability to maintain the specified position independently without auxiliary means.

### 4.5. Sensitivity Analysis of the Weight Coefficients in the Integral Control Quality Assessment

To assess the robustness of the obtained results, the quantitative sensitivity analysis of the integral control quality criterion $R_{Q}$ was conducted with respect to the choice of weight coefficients. Recall that the integral assessment is constructed as a weighted sum of the normalised metric values, reflecting both physiological and metric aspects of stability, comfort, and gait uniformity. The baseline weight values were selected based on expert ranking of the significance of each metric, followed by normalisation (see Section 3.4).

For the sensitivity analysis, the coefficient values were varied within ±20% (scale factor 0.2) using random multiplicative perturbations while preserving the total weight norm. For each weight sample, the integral scores and ranking of the alternatives were recalculated. A total of 1000 iterations were performed.

The summary stability statistics demonstrate a high degree of consistency; the mean Kendall’s coefficient between the original and varied rankings was 0.983 (standard deviation 0.029), which indicates high stability under weight variation. This means that the ranking of systems by control quality remains practically unchanged even with moderate distortions of the expert priorities.

**Figure 20:**
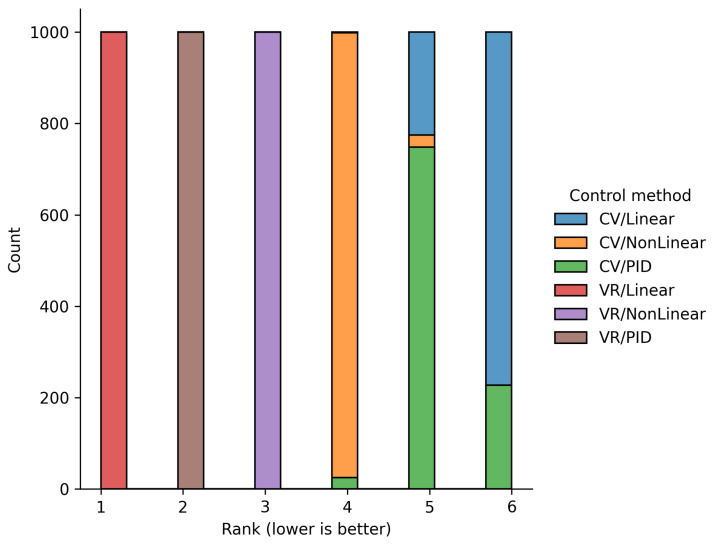
The results of the sensitivity analysis of the weight coefficients in the integral control quality assessment.

Figure 20 illustrates how frequently each configuration occupies a given rank position. The diagram shows that all functions retain their priorities, which is consistent with the results previously obtained using the expert-defined weight values.

Table 4 also presents the probability that, under weight variation, each alternative performs better or worse than its baseline position. For example, the probability of performance deterioration for CV/PID is 22.7%, while the probability of improvement is only 2.5%. For all three VR configurations, these probabilities are practically zero, further confirming the reliability of the obtained conclusions.

Thus, it can be concluded that the proposed integral criterion is resistant to reasonable variations in weight coefficients, and the ranking results for the control functions remain interpretable and well-founded.

## 5. Discussion

During this study, various control functions of the adaptive running platform were compared, and the influence of various aspects of the platform design on user movement was examined. The effectiveness of the control functions was assessed using the comprehensive integral criterion, which includes both subjective user assessments and a number of objective metrics that allow us to evaluate the positioning accuracy, smoothness of movement, duration and type of transition process occurring at the start of movement. Additionally, the pattern of user movement at the constant platform speed was analysed.

During the experiment, average optimal parameter values were selected for three different control functions: linear, nonlinear, and PID, the influence of these parameter values on various gait characteristics was determined. Analysis of these dependencies showed that in order to achieve the best results for each specific user, individual parameter selection is required. However, acceptable results can be obtained using average optimal values, or using these values as a starting point for individual adjustment within small limits, which will require significantly less time to adapt the system to a specific user. The linear function can be used as the most versatile and easy-to-configure control function, as it has shown one of the best results and is easier to configure than the others.

The comparison of the computer vision-based tracking system and MediaPipe Pose recognition technology with the reference system based on virtual reality trackers showed that tracking a person’s position using a single camera leads to worse results. However, this method is still applicable for this task. At the same time, the use of a computer vision system allows for a significantly greater amount of data about gait to be obtained, as the position of all key points of the body is tracked. This information can be used for a more complete analysis of gait during rehabilitation.

The comparison of different platform designs showed that the platform with an extended running surface length reduces the negative impact of the absence of handrails and allows users to move more freely at different speeds. The authors estimate the minimum acceptable length of the running platform surface to be between 1.6 and 1.8 m, taking into account the necessary safety margins at the beginning and end of the platform. Additionally, markings can be used to reduce the length of the platform, indicating the boundaries of the working area for the user.

During the experiments, the treadmill control system demonstrated stable behaviour in all operating modes. Properly configured control functions (linear, nonlinear, and PID with the selected coefficients) ensured process convergence: after the initial transient phase, the user consistently remained in the central zone of the treadmill belt without oscillations or divergence in platform speed (as illustrated in Figure 6). This stability is also facilitated by the limitation on maximum treadmill acceleration, which smooths abrupt changes.

Regarding system responsiveness, the full update cycle (from position reading to speed adjustment) directly depends on the frequency of the tracking system: 60 Hz for VR trackers and 30 Hz for the camera. Latency when using trackers is minimal—the total delay between position estimation and speed recalculation is 31.3 ± 0.72 ms. For the camera-based system, the delay is approximately 26.4 ± 0.48 ms (computation time), with an additional 8.46 ± 0.56 ms required to send the control command after detecting the person in the frame. These delays are within the acceptable range (below 100 ms), since the treadmill responds quickly enough for the user not to significantly shift relative to the working area. The slightly higher latency in the camera configuration may explain the somewhat lower smoothness and stability indicators observed with S2; however, overall, the system remains stable and controllable even at an update rate of 30 Hz.

The selected 60 s measurement interval with triple repetitions proved adequate during statistical analysis. Over 60 s, participants consistently reached the steady-state mode, and this time frame was sufficient to compute all metrics used in the study. Moreover, performing three repetitions per configuration yielded stable estimates without significantly increasing fatigue or compromising safety—a particularly important factor during the preliminary stage with extensive parameter tuning. Extending the duration or number of repetitions would have resulted in substantial additional time costs for evaluating and comparing the chosen control function parameters.

Safety was prioritised at all stages of the study. The maximum speed of both treadmills was limited to 2 m/s; platform No. 1 was additionally equipped with handrails and a safety harness. In the final implementation of platform No. 2, handrails and a similar safety mechanism will also be installed. The software included an automatic stop mechanism triggered when the user moved beyond the working area. During each trial, an operator was present, ready to immediately press the emergency stop button if necessary. Smooth acceleration/deceleration profiles and prior instruction further minimised the risk of losing balance. Across all 355 experiments, no incidents were recorded, confirming that the chosen experimental protocol provides sufficient safety.

The obtained results are important for the field of musculoskeletal rehabilitation. Smooth, low-oscillation speed control on a treadmill is critical for the tolerability and validity of clinical walking tests [44]. Visual target markings used in experiments to improve user positioning also have potential in the development of musculoskeletal rehabilitation systems. The reason for this is that treadmill training with visual biofeedback significantly improves balance and reduces the need for walking aids, which has also been confirmed by the results of experiments [45].

When comparing our results with existing speed control approaches, it should be noted that in studies involving force-measuring treadmills, the belt speed is often adjusted according to the vertical and horizontal components of the ground reaction force. Such controllers achieve low latency without the use of external trackers and accurately reproduce the calculated walking speed; however, they require a specialised hardware platform based on strain gauge sensors and do not address the issue of accessibility for widespread implementation [46]. Sensitivity studies of such controllers indicate that excessive sensitivity increases variability in both speed and step patterns, which is consistent with our observations. Under constraints of latency and tracking noise, simple linear rules tend to be practically preferable, as they provide predictable dynamics without excessive oscillations [47].

Overall, the direction of using strain gauge sensors as a source of information about a person’s position is highly promising, especially for musculoskeletal rehabilitation, as it allows reliable detection of each step and its associated force. Therefore, studying the feasibility of position estimation and platform control based on force-plate data will form a focus of our future research.

Existing studies on position-velocity control functions that use estimates of the person’s position and/or centre-of-mass velocity (including both marker-based and markerless systems) highlight the benefits of explicitly incorporating user speed into the control function, as well as the potential of predictive strategies to reduce delay, particularly during abrupt changes in gait tempo. Our results with linear and PID control functions confirm the following: under high-frequency, low-latency tracking (VR trackers), the linear law performs comparably to PID in terms of overall control quality, whereas under markerless tracking, the advantages of more complex laws are partially offset by limitations of the sensing channel (sampling rate, occlusions, lighting conditions) [31,48]. In this sense, the metric system and the integral criterion we propose can serve as a foundation for subsequent objective comparisons with algorithms offering enhanced predictive and state-analysis capabilities [49].

### Study Limitations

Consideration of all the limitations of the present study is necessary.

Despite the successful application of the proposed tracking systems under laboratory conditions, several constraints related to real-world implementation must be acknowledged. The S1 system based on VR trackers requires the installation of base stations and careful calibration before each session. In a rehabilitation centre, this entails additional effort from the staff, while for home use it presents difficulties for patients attempting to configure the equipment independently. The S2 system (MediaPipe Pose) is simpler and more accessible—requiring only a camera—yet its reliability depends on environmental conditions: lighting, background contrast, and absence of moving objects are all critical. Therefore, deployment of such a system either requires a controlled environment (for example, a dedicated equipped room) or the development of additional algorithms to compensate for these factors (background interference suppression, automatic recalibration, etc.). We acknowledge these limitations and assume that the practical value of the proposed system will be most evident in specially equipped rehabilitation facilities; for broad domestic use, alternative tracking solutions or controlled-environment adaptations will be necessary. Moreover, in selecting the model for the computer-vision-based tracking system, alternative options were not explored: for instance, the more computationally intensive OpenPose could potentially increase pose estimation accuracy, while lighter models could further reduce latency.

Another important limitation concerns markerless tracking systems: according to [20], the average joint angle estimation error can reach 11°. For high-precision clinical motion analysis, such an error is substantial—a 5–10° difference in knee flexion amplitude may correspond to a clinically significant discrepancy (as noted by [21]). In the context of the present study and the specific task of treadmill speed control, such high angular precision was unnecessary, since the analysis did not focus on segmental kinematics but only on the overall position of the body relative to the treadmill. However, for full clinical gait monitoring (e.g., assessing angular dynamics during rehabilitation), a more accurate model would be required. The position and velocity data obtained in this work are sufficient for adaptive control but do not substitute detailed 3D motion analysis. Hence, the proposed system is primarily designed for control purposes, whereas patient-progress diagnostics in angular parameters may require integration with higher-accuracy measurement systems.

This study was conducted on a limited sample of healthy volunteers (15 participants in total); therefore, the results cannot be directly generalised to other population groups. Individual differences in physical characteristics and gait patterns may affect the effectiveness of control algorithms, and the statistical significance of the observed trends cannot be confirmed given the small sample size. Even within this small group, such individual variations were already noticeable. For individuals with gait pathology (e.g., after neurological injuries or with balance disorders), greater positional fluctuations and higher variability in response to treadmill speed changes are expected. This implies that the control algorithms may require different tuning (smoother response, increased safety margins) to ensure comfort and safety for such users. Moreover, patients are likely to require continuous support (handrails, safety harnesses). We therefore recognise the absence of patient data as a limitation of the current study. In future research, we plan to involve a larger and more diverse group of participants, including individuals with gait impairments, to validate the general applicability and robustness of the findings and, if necessary, adapt the algorithms to patient-specific needs.

Finally, beyond the control laws considered here (linear, nonlinear, PID), other established methods are potentially applicable to adaptive treadmills—for instance, active disturbance rejection and state-filtering techniques [50] and neural-network-based regulators [51]. These approaches provide high robustness and/or predictive behaviour but require greater computational resources and complex system identification; in addition, the operation of neural-network-based control functions is not always transparent or stable [8]. In this work, we deliberately focused on transparent and easily tuneable control laws, which are critical for rehabilitation practice. At the same time, the proposed metric system and integral criterion can be directly employed for future comparisons with these advanced methods using the same hardware platform.

## 6. Conclusions

This paper presents the development and a comparison of control algorithms for an adaptive running platform for musculoskeletal rehabilitation tasks, taking into account various hardware configurations, motion tracking systems, and three control function options. A coordinated set of metrics is proposed and applied, reflecting both the stability of the user’s position and the nature of transitional processes, as well as subjective comfort. The metrics are aggregated into an integral criterion suitable for comparing controllers and adjusting parameters. Based on 355 tests, it has been shown that the linear function provides one of the best compromises between simplicity and quality when used with virtual reality trackers, while the nonlinear control function demonstrates the best performance when used with a computer vision system.

The experimental study consisted of two stages: preliminary (number of participants *n* = 5) for parameter selection, and main (*n* = 10) for comparison of functions and tracking systems; a total of 355 experiments were performed (three 60 s trials per configuration). The transition to a longer working area significantly reduces the possibility of the user going beyond the control limits and increases comfort.

The comparison of the two experimental platforms showed that increasing the length of the platform and removing the handrails creates a fundamentally different user experience. Platform No. 1 (1.73 m, with handrails and angle adjustment) and Platform No. 2 (2.58 m, without angle adjustment and handrails) differ in both kinematic limitations and permissible working area (1.50 m vs. 2.40 m), which directly affects position stability and the range of safe speeds. Based on a combination of metrics, the longer platform provides greater freedom of movement and mitigates the impact of the handrails’ absence. The minimum acceptable length of the track for rehabilitation tasks, based on the results of the experiments, is estimated at 1.7 m, taking into account the necessary safety zones at the edges. Based on the results of preliminary selection and basic comparison, practical bottom limits for treadmill dimensions have been formulated: the minimum acceptable length of the working area is 1 m for the linear function, 0.75 m for the nonlinear function, for the PID is about 1.1 m, taking into account the selected coefficients (*k_p_* = 0.5, *k_i_* = 0.5, *k_d_* = 0.5); a working area of 0.5 m is considered insufficient.

The tracking systems were tested on two configurations: S1 based on HTC Vive VR trackers and S2 based on the MediaPipe Pose computer vision system with conversion of relative coordinates into metric ones. Both systems convert the user’s position to a common metric system, but with identical control settings, S1 consistently outperforms S2 in terms of both the integral criterion and individual metrics of stability and smoothness. The specificity of S2 is manifested in greater sensitivity to control function settings; the best results were achieved with nonlinear control for S2, while the linear function showed a slight advantage for S1.

The limitations of the study include the small sample size, the fixed range of speeds, and the absence of a group with musculoskeletal impairments. These limitations do not diminish the value of the findings for engineering design but instead define the directions for future work: expanding the range of speeds and experimental scenarios, verifying repeatability, including clinical groups, and comparing the proposed system with predictive and multisensory control schemes based on the developed metric framework and integral criterion.

From a practical perspective, the results—in the form of the proposed parameterisation methodology, the set of metrics, and the comparative analysis protocol—enable reproducible comparison of configurations and facilitate the scaling of development across various setups and application conditions.

Thus, the study achieved all its goals and made recommendations on the minimum acceptable dimensions of the working area of treadmills, the selection of tracking systems, and control functions for musculoskeletal rehabilitation systems.

## Figures and Tables

**Table 1:** A summary analysis of the hardware capabilities of running platforms.

Characteristics	Platform No. 1	Platform No. 2
Track length, m	1.73	2.58
Platform working area ($L_{TR}$), m	1.50	2.40
Track width, m	0.90	0.90
Maximum acceleration, m/s^2^	2.2	2.5
Maximum speed, m/s	3	5.5
Handrails	yes	no
Angle adjustment	yes	no

**Table 2:** Weighting coefficient values for each metric.

$w_{i}$	Metric Name	Metric Weight
1	Rating	0.0737
2	$R_{SAO}$	0.1263
3	$R_{STO}$	0.1684
4	$R_{ZD}$	0.1263
5	$R_{ZFSMAE}$	0.0421
6	$R_{ZFMEAN}$	0.1579
7	$R_{ZMD}$	0.0211
8	$R_{ZAO}$	0.1263
9	$R_{ZTO}$	0.1579

**Table 3:** The results of statistical analysis based on the Mann–Whitney U test.

Metric	VR Linear/ NonLinear	VR Linear/PID	VR NonLinear/PID	CV Linear/ NonLinear	CV Linear/PID	CV NonLinear/PID
Rating	**0.0132**	**0.0108**	0.2046	0.1595	0.7427	0.5471
$R_{SAO}$	**0.0006**	0.7256	**0.0004**	0.1051	**0.0027**	0.0877
$R_{STO}$	0.6100	0.1715	0.0877	0.0575	**0.0004**	0.1023
$R_{ZD}$	**0.0000**	**0.0232**	**0.0000**	**0.0000**	0.0963	**0.0000**
$R_{ZFSMAE}$	0.8303	0.2581	0.2581	0.5395	0.4119	0.1907
$R_{ZFMEAN}$	0.7506	0.3403	0.3632	0.2905	0.7283	0.5395
$R_{ZMD}$	0.7958	0.4553	0.6843	**0.0003**	0.1373	**0.0351**
$R_{ZAO}$	**0.0000**	**0.0195**	**0.0000**	**0.0005**	0.0567	**0.0000**
$R_{ZTO}$	0.8303	0.0905	0.1537	**0.0327**	0.3871	**0.0103**

The *p*-values are indicated. The *p*-values less than 0.05 are highlighted in bold.

**Table 4:** Potential reprioritisation for each tracking-system/control-function combination.

Tracking System, Control Function	Probability of Improvement	Probability of Deterioration
(VR, Linear)	0.000	0.000
(VR, PID)	0.000	0.000
(VR, NonLinear)	0.000	0.000
(CV, NonLinear)	0.000	0.027
(CV, PID)	0.025	0.227
(CV, Linear)	0.227	0.000

## Data Availability

The datasets are available on request from the corresponding author only, as the data are sensitive and participants may be potentially identifiable.
